# Paraspinal Abscess in a Two-year-old Female

**DOI:** 10.5811/cpcem.2019.10.44857

**Published:** 2019-12-17

**Authors:** Rachel O’Donnell, Sean Sayani, Phillip Aguìñiga-Navarrete, Daniel Quesada, Kieron Barkataki, Madison Brooke Garrett

**Affiliations:** * Kern Medical Center, Department of Emergency Medicine, Bakersfield, California; † LAC + USC Medical Center, Department of Emergency Medicine, Los Angeles, California

## Abstract

A paraspinal abscess is an uncommon condition frequently diagnosed late due to equivocal symptoms, which can lead to increased morbidity and mortality. Commonly associated risk factors include prior invasive spinal procedures, diabetes mellitus, trauma, chronic steroid use, malnutrition, intravenous drug use and an immunocompromised state. Pediatric paraspinal abscesses are not well documented in the literature. We report a case of a two-year-old female presenting with fevers, lower back pain, and decreased oral intake ultimately diagnosed with isolated lumbar paraspinal abscess. The patient underwent an ultrasound-guided percutaneous drainage of the abscess, subsequently improving, and was discharged within 48 hours of presentation.

## INTRODUCTION

Due to the infrequency of paraspinal abscesses, no reliable figures on prevalence are documented in North America. Within developing nations, the incidence is roughly one in 100,000 – 250,000.^1^,^2^ The most common etiology is an abscess resulting from an invasive spinal procedure such as a lumbar puncture, epidural anesthesia, or both. Hematogenous, lymphatic and direct spread from adjacent sites have been reported, but are of greater rarity in the literature.^2^,^3^ Due to non-specific symptoms and unreliable expressions, pediatric patients are commonly diagnosed late in the disease process, which can increase morbidity and mortality.^3^ We present a rare case of pediatric paraspinal abscess, diagnosis and management.

## CASE REPORT

A two-year-old female born at term with no known medical history presented to the emergency department (ED) with her family complaining of fevers, right lower back pain, and abdominal pain for six days. The patient’s parents reported decreased oral intake, fussiness, and increased refusal to ambulate. Vitals were significant for a heart rate of 145 beats per minute and temperature of 39.8 degrees Celsius (103.6^o^ Fahrenheit). Physical examination was positive for a fussy, difficulty to console child who refused to stand or ambulate. A mild amount of swelling and erythema was observed on the right lower back, which was tender to palpation.

**Image 1:**
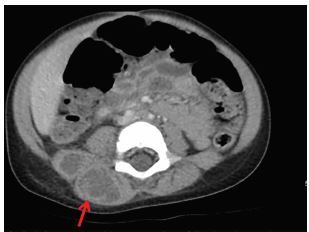
Axial computed tomography of the lumbar spine revealing areas of low density with some rim enhancement in the right posterior paraspinal muscles (arrow) consistent with abscess.

**Image 2:**
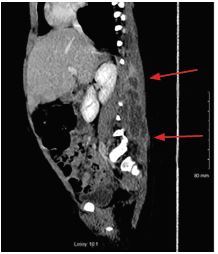
Sagittal computed tomography of the lumbar spine revealing a rim-enhancing paraspinal muscle abscess extending from lumbar vertebrae 1 (L1) to L5 (arrows).

Lab findings were significant for leukocytosis 15.2×10^9^ liters (L) (5.0×10^9^–11.0×10^9^ L), Lactic acidosis 4.6 millimoles (mmol) per L (2.0–4.0 mmol/L), elevated CRP (C-reactive protein) 7.4 milligrams per deciliter (mg/dL) (0.0–3.0 mg/dL) and ESR (erythrocyte sedimentation rate) 82 millimeters per hour (mm/hr) (3–13 mm/hr). Due to the clinical presentation and lab findings, a computed tomography (CT) of the abdomen and pelvis was ordered. The imaging was significant for multiloculated fluid collection in the right paraspinal musculature extending from lumbar vertebrate 1 (L1) to L5 without spinal, bony or canal involvement (Images 1 and 2). The patient was given intravenous (IV) fluids, IV vancomycin, and piperacillin/tazobactam; she subsequently had an ultrasound-guided percutaneous drainage of the abscess completed. Cultures of the abscess were positive for methicillin-susceptible *Staphylococcus aureus*. Blood cultures were negative. The patient had no resulting neurological sequelae, and was ambulating and eating within 24 hours. Her symptoms resolved and she was discharged with clindamycin palmitate within 48 hours of initially presenting to the ED.

## DISCUSSION

A paraspinal abscess is frequently diagnosed late in the disease process unless a physician has a high index of suspicion. Our patient had no identifiable etiology or risk factors to explain how the abscess might have developed. Paraspinal abscesses commonly occur in the mid-thoracic and lumbar spine, similar to our case.^3^ *S. aureus* accounts for up to 79% of isolated bacteria in pediatric cases, with Streptococcal and *Escherichia coli* infections also reported.^3^ Other possible organisms include fungi such as Candida and Cryptococcus species, although they are rare causes. Risk factors include previous spinal procedures, diabetes mellitus, trauma, immunocompromised state, chronic steroid use, malnutrition and IV drug use.^4^,^5^ Back pain and fever are common symptoms, seen in 50% of cases, and they were present in our case. Other symptoms include weakness, other neurological deficits, and death late in the disease course.^2^

**CPC-EM Capsule**

What do we already know about this clinical entity?

Paraspinal abscesses are rare in children. Usually these abscesses are due to invasive spinal procedures; less commonly, they are hematogenous or direct spread.

What makes this presentation of disease reportable?

This image demonstrates the rare finding of a paraspinal abscess in a pediatric patient with no identifiable risk factors.

What is the major learning point?

Paraspinal abscesses can present in the pediatric patient population, even without previous instrumentation or risk factors.

How might this improve emergency medicine practice?

Emergency physicians should consider broadening the differential to include paraspinal abscesses in pediatric patients presenting with non-specific symptoms.

CRP and ESR are both sensitive but not specific modalities that aid in diagnosis. A normal white blood cell count does not exclude the diagnosis. Procalcitonin recently has been shown to be helpful in diagnosis, especially when trying to discern if the organism is bacterial, but it has lower sensitivity than a CRP.^5^ Plain radiographs are not commonly used for diagnosis as pathology is difficult to visualize until it is late in the disase course (after 3–4 weeks). Some observable features include soft tissue swelling and eventual bony destruction.^2^ Magnetic resonance imaging is the gold standard for diagnostic imaging due to high sensitivity and specificity rates of 96% and 94%, respectively. CT imaging is used frequently due to cost and accessibility.^5^ Medical management using parenteral antibiotics can be initially attempted for smaller abscesses due to adequate penetration into paraspinal tissues, although larger abscesses may warrant percutaneous drainage. Surgical debridement should follow with failure of conservative management, neurological signs, spinal instability, and spinal lesions.^5^

## CONCLUSION

Paraspinal abscesses can be present with nonspecific symptoms. Pediatric patients can be more difficult to diagnose, as the child may be unable to effectively describe abdominal and back complaints, as in the presented patient. This case illustrates the importance of conducting a thorough history and physical examination with a wide differential diagnosis, especially in the pediatric population. Early diagnosis is imperative to prevent further progression of the disease course, and to reduce hospital stay.
